# Supplementary material for: Factors affecting men’s involvement in maternity waiting home utilization in North Achefer district, Northwest Ethiopia: A cross-sectional study
Source: PLoS One. 2022 Feb 10;17(2):e0263809. doi: 10.1371/journal.pone.0263809 (PMC8830716; doi:10.1371/journal.pone.0263809)
Supplement: S1 File — (PDF) [file pone.0263809.s002.pdf]

S1 File: Amharic language version questionnaire (አማርኛ መጠይቅ)

ባህር ዳር ዩኒቨርስቲ ህክምናና ጤና ሳይንስ ኮሌጅ ህብረተሰብ ትምህርት ቤት ስነ-ተዋልዶ ጤናና ስነ-ህዝብ ትምህርት ክፍል

ቀበሌ-------------------------ጎጥ------------------------------የመጠይቁ መለያ ከድ-------------------------------

አጠቃላይ <u>መረጃ መስጠት</u>

ጤና ይስጥልኝ! ስሜ (መረጃ ሰብሳቢዉ ስም) ______________________ይህ መጠይቅ በምዕ/ጎጃም ዞን ሰሜን አቸፈር ወረዳ የሚኖሩ የወንድ ትዳር አጋሮች፡ ሚስቶቻቸዉ ከእናቶች ማቆያ ክፍል ተኝተዉ የወለዱ፣ ከ 0-12 ወር እድሜ ህፃን ያላቸዉን ወንዶች በእናቶች ማቆያ ክፍል አጠቃቀም ላይ ያላቸዉን ተሳትፎ እና ተዛማጅ ምክኒያቶች ዳሰሳ የሚያደርግ መጠይቅ ነዉ፡፡ .እኔ የጥናቱ መረጃ ሰብሳቢ ነኝ፡፡ ይህን በማጥናት ለፖሊሲ አዉጪዎች እና ለለህክምና ባለሙያዎች በቂ የሆነ መረጃ በመስጠት በመረጃ ላይ የተመሰረተ ዉሳኔ እንዲወስኑ ያግዛል፡፡ እርስዎን ለቃለ መጠይቅ የመረጥንዎ በእድል እንጂ ሌላ ምንም አይነት ምክንያት የሌለን መሆኑን እንዲያውቁ እንፈልጋለን፡፡ ወደ ቃለ መጠይቁ ከመግባታችን በፊት ግን ስለ ጥናቱ አላማ እና አጠቃላይ ሁኔታ የማነብልዎትን እንዲያዳምጡኝና በጥናቱ ለመሳተፍ ፈቃደኛ መሆንዎንና አለመሆዎን አንድታረጋግጡልኝ እጠይቅዎታለሁ፡፡

<u>ፈቃደኛነትን መጠየቂያ ቅፅ</u>

የዚህ ጥናት አላማ የወንድ ትዳር አጋሮች ተሳትፎ በእናቶች ማቆያ ክፍል አጠቃቀም እና ተዛማጅ ጉዳዮች የሚያጠናና መሻሻል እና መስተካከል ያለባቸዉን ክፍተቶች የሚጠቁም ጥናት ነዉ፡፡ ጥናቱ የሚሰበሰበው በቃለ መጠይቅ ይሆናል፡፡ ቃለ መጠይቁ የሚወስደው ጊዜ ከ15 እስከ 20 ደቂቃ ይሆናል፡፡ ቃለመጠይቁ የግል ህይወት ጥያቄዎችን ያካትታል፡፡ ይህ የሚሰጡት መረጃ በሚስጥር እንደሚጠበቅ ላረጋግጥልዎት እወዳለሁ፡፡ እያንዳንዱ ተሳታፊ በሚስጥር ቁጥር ይለያል ስም አይጠቀስም፡፡ ቃለመጠይቁ በፈቃደኝነት ላይ የተመሰረተ ሲሆን ምንም አይነት ድጎማ የለውም፡፡ ጥያቄዎቹን ሙሉ በሙሉም ሆነ በከፊል የመመለስ መብት አለዎት፤ እንዲሁም ካልተመቸዎት በመሀል ቃለመጠይቁን ማቆም ይችላሉ፡፡ የእርስዎ መሳተፍ ወይም አለመሳተፍ አሁንም ሆነ ወደፊት እርሶም ሆነ ቤተሰብዎ በሚያገኙት አገልግሎት ላይ የሚያመጣው ጉዳት አይኖርም፡፡ በጥናቱ ዙሪያ ማንኛውም ጥያቄ ካለዎት ከዚህ በታች የተጠቀሱትን ሰዎች በሚፈልጉት ጊዜ ማነጋገር ይችላሉ፡፡ ጌታቸዉ አስማረ ኢሜል፤ gasmare35@gmail.com፣ ስልክ ቁጥር +251918315569

በጥናቱ ላይ ለመሳተፍ ፈቃደኛ ነዎት?

አመሰግናለሁ!!!

ሀ) አዎ   ለ) አይደለሁም

ተሳታፊው የቃለ ፈቃደኝነቱን መስጠቱን የሚረጋግጥ የጠያቂው ፊርማ

የጠያቂዉ ስም ___________ ___________ ፊርማ ___________ ቀን ----/-----/--------

የሱፐርቫይዘሩ ስም _________ ___________ ፊርማ ___________ ቀን ----/-----/--------

<u>የተዋቀረ መጠይቅ በአማርኛ</u>

***መመሪያ*:** የሚከተሉትን ጥያቄዎች ከጠየቁ በኋላ ምርጫ ለቀረበላቸው ጥያቄዎች መልሳቸውን ያከብቡ ምርጫ ላልቀረበላቸው ጥያቄዎች በተሰጠው ክፍት ቦታ ላይ መልሱን ይፃፉ፡፡

<u>ክፍል1.ማህበራዊ፣ ኢኮኖሚያዊና ዲሞግራፊያዊ ጥያቄዎች</u>

**ጥያቄዎች**	**አማራጭ መልስ**	**ይዘለል**
101.እድሜ (አሁን ላይ)	--	
102.ሀይማኖት	**1.** ኦርቶዶክስ<br>**2.** ሙስሊም<br>**3.** ፕሮቴስታንት<br>**4.** ሌሎች(ይጠቀሱ)----------------	
103.ብሄር	**1.** አማራ<br>**2.** ኦሮሞ<br>**3.** ትግሬ<br>**4.** አገዉ<br>**5.** ሌሎች(ይጠቀሱ)------------	

104. የትምህረት ደረጃ	**1.** ማነበብና መፃፍ የማይችል<br>**2.** ማንበብና መፃፍ ብቻ የሚችል<br>**3.** መጀመሪያ ደረጃ (1-8)<br>**4.** ሁለተኛ ደረጃ(9-12)<br>**5.** ዲፕሎማና ከዛ በላይ	
105. ስራ	**1** አርሶ አደር<br>**2** የግል ስራ<br>**3** ነጋዴ<br>**4** መንግስት ሰራተኛ<br>**5** ሌላ(ይጠቀስ)-------------	
106.ስንት ልጅ አለዎት**?**	--------------------------------	
107.በአካባቢዎ በብዛት የምትጠቀሙበት የመጓጓዣ አይነት ምን ምን ናቸዉ?	1. መኪና<br>2. ባጃጅ<br>3. ሞተር ብስክሌት<br>4. ብስክሌት<br>5. ሌላ(ጥቀሱ)--------------	

ክፍል ሁለት፡ የቤተሰቡን ገቢ የሚጠይቁ ጥያቄዎች

ጥያቄዎች	አማራጭ መልስ	ይዘለል

201.ቤተሰቡ የመጠጥ ዉሃ የሚያገኘዉ ከየት ነዉ?	**<u>የቧንቧ ዉሃ</u>**<br>1. ከመኖርያ ቤቱ ዉስጥ ያለ<br>2. ከግቢዉ ዉስጥ ያለ<br>3. ከግቢዉ ዉጭ ያለ<br>4. በመወዝወዝ የሚቀዳ<br>**<u>የዉሃ ገድጓድ</u>**<br>**1.** የተከለለ<br>**2.** ያልተከለለ<br>**<u>የምንጭ ዉሃ</u>**<br>1. የተከለለ የምንጭ ዉሃ<br>2. ያልተከለለ የምንጭ ዉሃ<br>3. የዝናብ ዉሃ<br>4. በመኪና የዉሃ ማጓጓዣ ታንከር የሚመጣ ዉሃ<br>**<u>ከርሰምድር ዉሀ፣ወንዝ፣ኩሬ፣ሀይቅ ዉሀ</u>**<br>1. የመስኖ ዉሀ<br>2. የታሸገ ዉሀ<br>3. ሌሎች (ጥቀሱ)	
202.የቤተሰቡ አባላት የሚጠቀሙበት መፀዳጃ ቤት ምን አይንት ነዉ?	**<u>በዉሃ የሚሰራ መፀዳጃ ቤት</u>**<br>1. የፍሳሽ ማስወገጃ ቱቦ ያለዉ<br>2. የፍሳሽ ማጠራቀሚያ /ታንክ ያለዉ<br>3. ፍሳሹ ወደ ሽንት ቤት ጉድጓድ የሚፈስ መፀዳጃ ቤት	

<table>
<tr>
<td></td>
<td>4. ፍሳሹ ወደ ሌላ ቦታ የሚፈስ
5. ፍሳሹ የት ቦታ እንደሚፈስ የማይታወቅ

<u>ያለዉሃ የሚሰራ መፀዳጃ ቤትት/የሽንት ቤት ጉድጓድ</u>

1. የተሻሻለ የንፋስ መግቢያ ያለዉ የሽንት ቤት ጉድጓድ
2. ክዳን ያለዉ ሽንት ቤት ጉድጓድ
3. ክዳን የሌለዉ ሽንት ቤት ጉድጓድ
4. ክፍትና ሰፋ ያለ የሽንት ቤት ጉድጓድ
5. ወደ ብስባሽ የሚቀየር መፀዳጃ ቤት
6. የባልዲ መፀዳጃ ቤት
7. የተንጠለጠለ ሽንት ቤት
8. መፀዳጃ ቤት የሌለዉ ቁጥቋጦ ዉስጥ/ሜዳ ላይ መፀዳዳት
9. ሌላ(ጥቀሱ)-------------</td>
<td></td>
</tr>
<tr>
<td>203. ከሚከተሉት የቤት እንስሳቶች ዉስጥ ቤተሰቡ ምን ያህሉን አለዉ?
የወተት ላም፤በሬ ወይም ኮርማ?

ፈረስ ፤አህያ፤በቅሎ?

ግመል?

ፍየል?

በግ?</td>
<td>
<table>
<tr><td>አይነት</td><td>አለ</td><td>የለም</td></tr>
<tr><td>የወተት ላም፤በሬ ወይም ኮርማ?</td><td></td><td></td></tr>
<tr><td>ፈረስ ፤አህያ፤በቅሎ?</td><td></td><td></td></tr>
<tr><td>ግመል?</td><td></td><td></td></tr>
<tr><td>ፍየል?</td><td></td><td></td></tr>
<tr><td>በግ?</td><td></td><td></td></tr>
<tr><td>ዶሮ?</td><td></td><td></td></tr>
</table>
</td>
<td></td>
</tr>
</table>

ዶሮ?<br><br>የንብ ቀፎ?	የንብ ቀፎ?			
204.ቤትዎ ዉስጥ ከዚህ በታች የተዘረዘሩት አሉ?<br>ኤሌክትሪክ?<br>ሰዓት?<br>ሬድዮ?<br>ቴሌቭዥን?<br>ተንቀሳቃሽ ስልክ?<br>የማይንቀሳቀስ ስልክ?<br>ፍሪጅ?<br>ወንበር?<br>ጠረንጴዛ?<br>አልጋ?<br>ኤሌክትሪክ ምጣድ?<br>ኩራዝ?<br>ፋኑስ?	እቃ	አለ	የለም	
	ኤሌክትሪክ?			
	ሰዓት?			
	ሬድዮ?			
	ቴሌቭዥን?			
	ተንቀሳቃሽ ስልክ?			
	የማይንቀሳቀስ ስልክ?			
	አልጋ?			
	ኩራዝ?			
	ፍሪጅ?			
	ወንበር?			
	ፋኑስ?			
205.የቤትዎ ወለል በዋናነት የተሰራዉ ከምን ነዉ? (በማየት ይምላ)	**የተፈጥሮ ወለል**<br>1. አሸዋ/አፈር<br>2. እበት			

<table>
<tr><td rowspan="1"></td><td colspan="3"><u>የተጀመረ/ያላለቀ ወለል</u>
1. ቄጠማ/ሸንበቆ
2. የእንጨት ንጣፍ
<u>ያለቀ ወለል</u>
1. የተጠላለፈ የወለል ጣዉላ
2. እንጨት
3. ሴራሚክ
4. የስሚንቶ ጡብ
5. ስጋጃ/ምንጣፍ
6. ሌላ(ጥቀስ)---------</td><td></td></tr>
<tr><td>206.ከቤተሰቡ አባላት ዉስጥ ለግብርና ስራ የሚያገለግል መሬት ያለዉ አለ?</td><td colspan="3">1. አዎ
2. አይደለም</td><td>አይደለም ከሆን ወደ ጥያቄ 208 ዝለል</td></tr>
<tr><td>207.መልሱ አዎ ከሆነ በአካባቢዎ መለኪያ ምን ያክል ነዉ?</td><td colspan="3">በቃዳ/በገመድ---------------------------</td><td></td></tr>
<tr><td>208. ቤትዎ ዉስጥ ለቤተሰብዎ ምኝታ አገልግሎት የሚዉሉ ክፍሎች ምን ያህል ናቸዉ?</td><td colspan="3">-----------------------------------</td><td></td></tr>
<tr><td rowspan="4">209.ከቤተሰቡ አባላት ዉስጥ ከዚህ በታች የተዘረዘሩት አላችሁ?
ብስክሌት?
ሞተር ሳይክል?</td><td>አይነት</td><td>አለ</td><td>የለም</td><td rowspan="4"></td></tr>
<tr><td>ብስክሌት?</td><td></td><td></td></tr>
<tr><td>ሞተር ሳይክል?</td><td></td><td></td></tr>
<tr><td>በእንስሳት ሚጎተት ጋሪ?</td><td></td><td></td></tr>
</table>

በእንስሳት ሚጎተት ጋሪ?<br><br>መኪና ወይም ተሳቢ?<br><br>ሞተር የሌለዉ ጀልባ?<br><br>ባለሞተር ጀልባ?	ሞተር የሌለዉ ጀልባ?			
	ባለሞተር ጀልባ?			
	መኪና ወይም ተሳቢ?			

ክፍል ሶስት፡የወንድ የትዳር አጋሮችን ተሳትፎ የሚመለከቱ ጥያቄዎች

መመሪያ: የሚከተሉትን ጥያቄዎች ከጠየቁ በኋላ ምርጫ ለቀረበላቸው ጥያቄዎች መልሳቸውን ያክብቡ ምርጫ ላልቀረበላቸው ጥያቄዎች በተሰጠው ክፍት ቦታ ላይ መልሱን ይፃፉ፡፡

ጥያቄዎች	አማራጭ መልስ	ዝለል
301. ባለቤትዎ የአሁኑን ህፃን ስትወልድ፤ እናቶች ማቆያ ክፍል ቆይታ እንድትወልድ ዉሳኔ ወስነዉ ነበር?	1. አዎ<br>2. አይደለም	
302. ባለቤትዎ የአሁኑን ህፃን ስትወልድ፤ ወደ እናቶች ማቆያ ክፍል ለማረፍ የሄደችዉ ከእርስዎ ጋር ነበር?	1. አዎ<br>2. አይደለም	
303. ባለቤትዎ የአሁኑን ህፃን ስትወልድ፤ ወደ እናቶች ማቆያ ክፍል ስትሄድ ወይም እዚያዉ እያለች የገንዘብ ድጋፍ አድርገዉላት ነበር?	1. አዎ<br>2. አይደለም	
304. ባለቤትዎ የአሁኑን ህፃን ስትወልድ፤ እናቶች ማቆያ ክፍል በነበረች ጊዜ ለባለቤትዎና አብረዋት ላሉ ዘመዶቿ ምግብ ያቀርቡ ነበር?	1.አዎ<br>2.አይድለም	

305. ባለቤትዎ የአሁኑን ህፃን ስትወልድ፤ እናቶች ማቆያ ክፍል በነበረች ጊዜ ቤቱን ወይም ልጆችን እንክብካቤ ያደርጉ ነበር?	1.አዎ<br><br>2.አይድለም	መልሱ አይደለም ከሆነ ወደ ጥያቄ ቁጥር 307 ዝለል
306.ለጥያቄ ቁጥር 305 መልሱ አዎ ከሆነ፤ምን አይነት እንክብካቤ ያደርጉ/ላቸዉ ነበር?	1. ምግባቸዉን መዘገጀት<br>2. ልብሳቸዉንና ሰዉነታቸዉን ማጠብ<br>3. ሌሎች (ይጠቀሱ)………………	
307. ለጥያቄ ቁጥር 305 መልሱ አይደለም ከሆነ፣ማን ነበር እንክብካቤ ሚያደርገዉ/ላቸዉ?	1. ቤተሰቦቼ<br>2. ታላቋ ሴት ልጄ<br>3. ጎረቤቶቼ<br>4. የአካባቢያችን የሴቶች የልማት ቡድኖች<br>5. ሌሎች(ይጠቀሱ)----------	
308. ባለቤትዎ የአሁኑን ህፃን ስትወልድ፤ ወደ እናቶች ማቆያ ክፍል ለማረፍ ስትሄድ መጓጓዣ አመቻችተዉላት ነበር?	1.አዎ<br><br>2.አይደለም	

ክፍል አራት ፡ስለሴት ትዳር አጋሮቻቸዉ ከወሊድና ከእርግዝና ጋር የተያያዙ ጥያቄዎች

መመሪያ: የሚከተሉትን ጥያቄዎች ከጠየቁ በኋላ ምርጫ ለቀረበላቸው ጥያቄዎች መልሳቸውን ያክብቡ፡፡ ምርጫ ላልቀረበላቸው ጥያቄዎች በተሰጠው ክፍት ቦታ ላይ መልሱን ይፃፉ፡፡

**ጥያቄ**	**አማራጭ መልስ**	**ዝለል**

401. አሁን ከተወለደዉ ህፃን በፊት ባለቤትዎ ወልዳ ታዉቃለች?	1.አዎ<br><br>2.አይድለም	መልሱ አይደለም ከሆነ ወደ ጥያቄ 408 ዝለል
402. ባለቤትዎ ከአሁኑ ህፃን በፊት የተወለደዉን/ችዉን ልጅ ጤና ተቋም ነበር የወለደችዉ/ቻት?	1.አዎ<br><br>2.አይድለም	
403. ባለቤትዎ ይህን/ችን ህፃን ከመዉለዷ በፊት ከወሊድ ጋር የተያያዘ ችግር አጋጥሟት ነበር?	1. አዎ<br>2. አይድለም<br>3. አላስታዉሰዉም	መልሱ አይደለም/አላስ ታዉሰዉም ከሆን ወደ ጥያቄ 405 **ዝለል**
404. መልሱ አዎ ከሆነ፣ምን አይነት ችግር ነበር ያጋጠማት?	1. ጊዜዉ ያልደረሰ ምጥ<br>2. ምጥ ሳይጀምር የእንሽርት ዉሃ መፍሰስ<br>3. ደም መፍሰስ<br>4. ለረጅም ጊዜ የቆየ ምጥ<br>5. 5. ሌሎች(ጥቀሱ)	
405.ባለቤትዎ ይችን/ህን ህፃን ከመዉለዷ በፊት ሙቶ የተወለደ/ች ህፃን ነበር?	1.አዎ<br><br>2.አይደለም	

406.የአሁኑን ህፃን ስትወልድስ ለምን ያህል ጊዜ ተኝታ ቆየች	በቀን------------------------------------	
407. የአሁኑ ህፃን ሲወለድ ባለቤትዎ የነብሰ-ጡር ክትትል ታደርግ ነበር?	1.አዎ<br>2.አይድለም	መልሱ አይደለም ከሆነ ወደ ቀጣዩ ክፍል ዝለል
408. መልሱ አዎ ከሆነ፣እርስዎ አብረዋት ይሄዱ ነበር?	1.አዎ<br>2.አይድለም	
409. መልሱ አዎ ከሆነ፣ስለእናቶች ማቆያ የምክር አገልግሎት አግኝተዉ ነበር?	1.አዎ<br>2.አይድለም	

ክፍል አምስት፡ጤና ተቋሙን የሚመለከቱ ጥያቄዎች

***መመሪያ*:** የሚከተሉትን ጥያቄዎች ከጠየቁ በኋላ ምርጫ ለቀረበላቸው ጥያቄዎች መልሳቸውን ያክብቡ፡፡ ምርጫ ላልቀረበላቸው ጥያቄዎች በተሰጠው ክፍት ቦታ ላይ መልሱን ይፃፉ፡፡

**ጥያቄ**	**አማራጭ መልስ**			**ዝለል**
501. እናቶች ማቆያ ክፍል ዉስጥ ከዚህ በታች የተዘረዘሩት መሰረታዊ የሆኑ ማህበራዊ አገልግሎቶች ነበሩ?<br>መተኛ ቦታ?<br>አልጋ?	አይነት	አለ	የለም	
	መተኛ ቦታ?			
	አልጋ?			
	ትራስ?			

ትራስ?<br>ብርድ ልብስ?<br>ዉሃ?<br>ሽንት ቤት?<br>ተጨማሪ ክፍል ለሌላ ትንሽ ልጅ/ዘመድ መተኛ ቦታ?<br>መዝናኛ አገልግሎት ሬዲዮ፣ቴሌቪዢን?<br>የራሱ ማብሰያ ክፍል?<br>ሻወር ቤት?	ብርድ ልብስ?			
	ዉሃ?			
	ሽንት ቤት?			
	ተጨማሪ ክፍል ለሌላ ትንሽ ልጅ/ዘመድ መተኛ ቦታ?			
	መዝናኛ አገልግሎት ሬዲዮ፣ቴሌቪዢን?			
	የራሱ ማብሰያ ክፍል?			
	ሻወር ቤት?			
502.ከወሊድ ጋር በተያያዘ ችግር ቢፈጠር እናትየዋን ሪፈር ለማለት የአምቡላንስ አገልግሎት አለ?	1. አዎ<br>2. አይድለም			
503. ጤና ባለሙያዎች በየቀኑ ማታ፣ቅዳሜና እሁድ፣እንዲሁም በዓላት ቀናትን ጨምሮ እየዞሩ ማየት፣ወሳኝ ምልክቶችን መለካት፣የፅንሱን ሁኔታ የፅንስ መከታተያ ቻርት በማዘጋጀት መከታተል አይነት አገልግሎቶችን ያደረጉላት ነበር?	1. አዎ<br>2. አይድለም			

504. ከቤትዎ ተነስተዉ እናቶች ማቆያ ክፍል ለመድረስ ምን ያህል ሰዓት በእግር ጉዞ ይጨርሳል?	---------------------------------------	

ክፍል ስድስት ፆታ /ስርዓተ ፆታ ጋር የተያያዙ ጥያቄዎች

መመሪያ: የሚከተሉትን ጥያቄዎች ከጠየቁ በኋላ ምርጫ ለቀረበላቸው ጥያቄዎች መልሳቸውን ያክብቡ፡፡ ምርጫ ላልቀረበላቸው ጥያቄዎች በተሰጠው ክፍት ቦታ ላይ መልሱን ይፃፉ፡፡

**ጥያቄ**	**አማራጭ መልስ**	**ዝለል**
601. ምን አይነት ፆታን መሰረት ያደረጉ ምክኒያቶች ናቸዉ የወንድ የትዳር ዐጋሮችን በእናቶች መቆያ ክፍል አጠቃቀም ላይ ዉሳኔ እንዳይወስኑ የሚያደርጉት?(ከአንድ በላይ መልስ መምረጥ የቻላል)	1. ልጅ መዉለድ የሴቶች ጉዳይ ስለሆነ የወንዶች ተሳትፎ አያስፈልግም<br>2. ልጅ መዉለድ ተፈጥሯዊ ስለሆነ ብዙም ትኩረት በወንዶች አይሰጠዉም<br>3. ለወሊድ ሚስቶችን ወደ እናቶች ማቆያ ክፍል ይዞ መሄድ የሌሎች ሴቶች ሃላፊነት ነዉ	
602.አሁን አብራዎት ካለችዉ ባለቤትዎ በተጨማሪ ሌላ ሚስት አለዎት?	1. አዎ<br>2. አይደለም	
603. በእርስዎ ቤተሰብ ዉስጥ ዉሳኔ ሚያስፈልጋቸዉ ጉዳዮች ሲያጋጥሙ በአብዛኛዉ የሚወስን ማን ነዉ?	1. እኔ<br>2. ባለቤቴ<br>3. ሁላችንም በእኩልነት ተወያይተን ነዉ ምንወስነዉ<br>4. ሌላ(ይጠቀስ)---------------------	

ክፍል ሰባት፡ ከእናቶች ማቆያ ክፍል ጋር በተያያዘ እዉቀትን እና አመለካከትን የሚጠይቁ ጥያቄዎች

***መመሪያ*:** የሚከተሉትን ጥያቄዎች ከጠየቁ በኋላ ምርጫ ለቀረበላቸው ጥያቄዎች መልሳቸውን ያክብቡ፡፡ ምርጫ ላልቀረበላቸው ጥያቄዎች በተሰጠው ክፍት ቦታ ላይ መልሱን ይፃፉ፡፡

ጥያቄ	አማራጭ መልስ	ዝለል
701. ስለ እናቶች ማቆያ ክፍል መረጃዉን ከየት ነዉ ያገኙት?<br>(ከአንድ በላይ መልስ መምረጥ የቻላል)	1. ከጓደኛ<br>2. ከመገናኛ ብሁሀን(ሬዲዮ፣ቴሌቪዥን…)<br>3. ከባለቤቴ<br>4. ከሀይማኖት መሪዎች<br>5. ከጤና ባለሙያዎች<br>6. ሌሎች ካሉ ግለፁ	
702. ምን አይነት እናቶች ናቸዉ እናቶች ማቆያ ክፍል ገብተዉ የሚተኙት?(ከአንድ በላይ መልስ መምረጥ የቻላል)	1. በጣም እሩቅ ቦታ ወይም ለአንቡላስ አስቸጋሪ ከሆነ ቦታ የሚኖሩ እናቶች<br>2. ›38 ወር የምላቸዉና ለመዉለድ ሁለት ሳምንት የቀራቸዉ<br>3. ከአሁን በፊት በእርግዝና ወይም በወሊድ ጋር የተያያዘ ችግር ማለትም ሙቶ የተወለደ ህፃን፣የማህፃን በር ጫፍ መሰንጠቅ፣ጊዜዉ ሳይደርስ ምጥ መጀመር የመሳሰሉት ችግሮች ያሉባት እናት<br>4. ጤና ባለሙያዉ ብትተኛ ብሎ ከወሰነ	

	5. አላዉቅም<br>6. ሌሎች(ይጠቀሱ)-------------	
703. የእናቶች ማቆያ ክፍልን መጠቀም ለምን ይጠቅማል?(ከአንድ በላይ መልስ መምረጥ የቻላል)	1. በሰለጠነ ባለሙያ የወሊድ አገልግሎት ለማግኘት<br>2. ድንገተኛ ህክምና በሚያስፈልግበት ጊዜ መዘግየት እንዳይኖር ያደርጋል<br>3. ለእናትየዋ እና ለልጇ አፋጣኝ ህክምና ለመስጠት<br>4. አላዉቅም<br>5. ሌሎች(ይጠቀሱ)-----------------	
704. የእናቶችን ማቆያ ክፍል መጠቀም ሌሎች የእናቶች ጤና አገልግሎቶችን ለመጠቀም ያግዛል ብለዉ ያስባሉ?	1. አዎ<br>2. አይደለም	መልሱ አይደለም ከሆነ ወደ ጥያቄ 706 ዝለል
705. መልሱ አዎ ከሆነ ምን ምን አገልግሎቶችን ለማግኘት ያግዛል?(ከአንድ በላይ መልስ መምረጥ የቻላል)	1. የክትባት አገልግሎት<br>2. ወዲያዉኑ የእናት ጡት መመገብ እንዲጀምር<br>3. የአመጋገብ ምክር አገልግሎት<br>4. የቤተሰብ ምጣኔ አገልግሎት<br>5. ሌሎች(ይጠቀሱ)--------------	
706. የእናቶች ማቆያ ክፍል ጠቀሜታዉ ለማን ነዉ ብለዉ ያስባሉ?	1. ለእናትየዋ ብቻ<br>2. ለህፃኑ ብቻ<br>3. ለሁሉም ለእናትየዋም እና ለልጁም	

707.የእናቶች ማቆያ ክፍልን መጠቀም በምጥና በወሊድ ጊዜ ችግር ሲያጋጥም ወዲያዉኑ ችግሩን ለመለየት ይጠቅማል::	1. በጣም እስማማለሁ<br>2. እስማማለሁ<br>3. ገለልተኛ ነኝ<br>4. አልስማማም<br>5. በጣም አልስማማም	
708.በእናቶች ማቆያ ክፍል ዉስጥ የጤና ባለሙያዎች አገልግሎት አሰጣጥ እና አቀራረብ ጥሩ አይድለም::	1. በጣም እስማማለሁ<br>2. እስማማለሁ<br>3. ገለልተኛ ነኝ<br>4. አልስማማም<br>5. በጣም አልስማማም	
709.የእናቶች ማቆያ ክፍልን መጠቀም የጨቅላ ህፃናትን ሞት ይቀንሳል::	1. በጣም እስማማለሁ<br>2. እስማማለሁ<br>3. ገለልተኛ ነኝ<br>4. አልስማማም<br>5. በጣም አልስማማም	
710.ወንድ ልጅ ሚስቱን ወደ እናቶች ማቆያ ክፍል ይዞ ከሄደ “ሴታሴት” ነዉ::	1. በጣም እስማማለሁ<br>2. እስማማለሁ<br>3. ገለልተኛ ነኝ<br>4. አልስማማም<br>5. በጣም አልስማማም	
711. የእናቶችን ማቆያ ክፍልን መጠቀም ሌሎች የእናቶች ጤና አገልግሎቶችን ለመጠቀም ያግዛል::	1. በጣም እስማማለሁ<br>2. እስማማለሁ	

	3. ገለልተኛ ነኝ<br>4. አልስማማም<br>5. በጣም አልስማማም	
712. የእናቶች ማቆያ ክፍል መጠቀምን አልደገፈዉም ምክኒያቱም እናቶች በምጥና በወሊድ ወቅት የሚሞቱት በሰይጣን ነዉ::	1. በጣም እስማማለሁ<br>2. እስማማለሁ<br>3. ገለልተኛ ነኝ<br>4. አልስማማም<br>5. በጣም አልስማማም	
713.በአብዛኛዉ እናቶች ማቆያ ክፍል ሚተኙ ሴቶች ሰነፎች ናቸዉ::	1. በጣም እስማማለሁ<br>2. እስማማለሁ<br>3. ገለልተኛ ነኝ<br>4. አልስማማም<br>5. በጣም አልስማማም	
714.የእናቶች ማቆያ ክፍልን መጠቀም እናቶች በህይወት እንዲቆዩ ያደርጋል::	1. በጣም እስማማለሁ<br>2. እስማማለሁ<br>3. ገለልተኛ ነኝ<br>4. አልስማማም<br>5. በጣም አልስማማም	

አመሰግናለሁ!!!
