# Supplementary material for: Factors affecting men’s involvement in maternity waiting home utilization in North Achefer district, Northwest Ethiopia: A cross-sectional study
Source: PLoS One. 2022 Feb 10;17(2):e0263809. doi: 10.1371/journal.pone.0263809 (PMC8830716; doi:10.1371/journal.pone.0263809)
Supplement: S2 File — (PDF) [file pone.0263809.s003.pdf]

S2 File: English language version questionnaire

Bahir Dar University, College of Medicine and Health Sciences, School of Public Health, Department of Reproductive Health and Population Studies

Kebele-------------------------

Got-----------------------

Code of respondent-----------------------------

Information Sheet

This a questionnaire on assessment of men's involvement in maternity waiting home utilization and its associated factors in North Achefer district, West Gojjam zone, Northwest Ethiopia.

How are you? My name is (Name of the data collector) ____________________. We are conducting health research on assessment on male partners' involvement in maternity waiting home utilization and its associated factors among households who have children age 0-12 months and whose spouse utilize MWHs for the current child.

This is beneficial to identify areas of improvement in the male partners' involvement in maternity waiting home utilization and its associated factors and highlighting the need for corrective actions. By doing this we will provide sufficient information for policy makers and clinicians so that they could make informed decision. I would like to inform you that you are chosen to be interviewed by chance. Before, we go to the interview, I will request you to listen carefully to what I am going to read to you about the purpose and general condition of the study and tell me whether you agree or disagree to participate in this study.

Consent form

The purpose of this study is to identify areas of improvement in the male partners' involvement in maternity waiting home utilization and its associated factors and highlighting the need for corrective actions. The study will be conducted through interviews. The interview will only take about 15-20 minutes of your time. At the end, it is hoped that the information you give us could help to improve male partners' involvement in maternity waiting home utilization and hence decrease maternal and neonatal mortality. The interview may involve private life questions. I would like to assure you that this privacy should strictly be kept confidential. A code number will be used to identify every participant and no name will be used. The interview is voluntary and there will not be any incentives. You have the right to respond or not respond to the all or some questions. You can also stop the interview in between if you are not interested. Your participation or non-participation, or refusal to respond to the questions will have no effect now or in the future on services that you or any member of your family may receive from service providers or health facilities.

If you have any question you may contact: Getachew Asmare, Mobile phone: *+251918315569, Email gasmare35@gmail.com*

Are you willing to participate in this study?

1. Yes 2. No

Thank you!!

If the study subject agrees to participate in the study, start the interview.

Interviewer signature certifying that informed consent has been given verbally by the respondent.

Name of the interviewer_________________ Signature ______________Date _______________

Name of supervisor ___________________ Signature________________ Date______________

Sections the questionnaire

General instruction: Ask the following questions and then circle their answer on the response column if it is a choice question or write their answer on the blank space if it is an open ended question.

Section 1. Socio-demographic and economic factors

Questions	Responses	Skip to question
101.Age (current age) in years	---	
102.Religion	1. Orthodox<br>2. Muslim<br>3. Protestant<br>4. Other(specify)-------------	
103.Ethnicity	1. Amhara<br>2. Oromo<br>3. Tigre<br>4. Agew<br>5. Others(specify) -------------	
104. Educational status	1. Can't read and write<br>2. Read and writes<br>3. primary education(1-8)<br>4. secondary education(9-12)<br>5. Diploma and above	

105. Occupation	1. Farmer<br>2. Private business<br>3. Merchant<br>4. Government employee<br>5. Other(specify)	
106. How many children do you have?	---------------------------------	

Section 2 Question related to wealth status

**Questions**	**Responses**	**Skip to question**
201. What is the main source of drinking water for members of your household?	**piped water**<br>1. piped into dwelling<br>2. piped into compound<br>3. piped outside compound<br>4.tube well or borehole<br>**dug well**<br>5. protected well<br>6. unprotected well<br>**water from spring**<br>8. protected spring	

	9. unprotected spring<br><br>10. rainwater<br><br>11. tanker truck<br><br>**surface water (river/dam/**<br><br>**lake/pond/stream/canal/**<br><br>12. irrigation channel)<br><br>13. bottled water<br><br>14. other(specify) ____________________	
202. What kind of toilet facility do members of your household usually use?	**flush or pour flush toilet**<br><br>1. flush to piped sewer system<br>2. flush to septic tank<br>3. flush to pit latrine<br>4. flush to somewhere else<br>5. flush, don't know where<br><br>**pit latrine**<br><br>1. ventilated improved pit latrine (VIP)<br>2. pit latrine with slab<br>3. pit latrine without slab/<br>4. open pit	

	5. composting toilet<br>6. bucket toilet<br>7. hanging toilet/hanging latrine<br>8. no facility/bush/field<br>9. other(specify)____________________			
	**Item**	**Yes**	**No**	
203. How many of the following animals do this household own?	Milk cows, oxen or bulls?			
	Horses, donkeys, or mules?			
	Camels?			
	Goats?			
	Sheep?			
	Chickens?			
	Beehives?			
204.Does your household have:	**<u>Item</u>**	**<u>yes</u>**	**<u>No</u>**	
Electricity?	Electricity?			
A watch?	A watch?			

A radio?<br><br>A television?<br><br>A mobile telephone?<br><br>A non-mobile telephone?<br><br>A refrigerator?<br><br>A table?<br><br>A chair?<br><br>A bed?<br><br>An electric mitad?<br><br>A kerosene lamp/pressure lamp?	A radio?			
	A television?			
	A mobile telephone?			
	A non-mobile telephone?			
	A refrigerator?			
	A table?			
	A bed?			
	An electric mitad?			
	A kerosene lamp/pressure lamp?			
205. Main material of the floor.	**natural floor**<br><br>1. earth/sand<br>2. dung<br><br>**rudimentary floor**<br><br>1. wood planks<br>2. reed/bamboo<br><br>**finished floor**			

<table>
<tr><td></td><td>1. parquet or polished
2. wood
3. ceramic tiles
4. cement/bricks
5. carpet
6. other(specify)______________________
_</td><td></td></tr>
<tr><td>206. Does any member of this household own any land that can be used for agriculture?</td><td>1. yes
2. no</td><td>If no pass to question 208</td></tr>
<tr><td>207. How many (LOCAL UNITS) of agricultural land do members of this household own?</td><td>Local unit---</td><td></td></tr>
<tr><td>208. How many rooms in this household are used for sleeping?</td><td>--</td><td></td></tr>
<tr><td>209.Does any member of this household own:

A bicycle?

A motorcycle or motor scooter?</td><td><table><tr><td>Item</td><td>yes</td><td>No</td></tr><tr><td>A bicycle?</td><td></td><td></td></tr><tr><td>A motorcycle or motor scooter?</td><td></td><td></td></tr></table></td><td></td></tr>
</table>

An animal-drawn cart?<br>A car or truck?<br>A boat without a motor?<br>A boat with a motor?	An animal-drawn cart?			
	A car or truck?			
	A boat without a motor?			
	A boat with a motor?			

Section 3 male partners' involvement in maternity waiting home utilization

**Question**	**Response**	**Skip to question**
301. Did you decide for your spouse to use MWHs for the current child?	1.Yes<br>2.No	
302. Did you accompany your wife while she went to MWH for the current child?	1.Yes<br>2.No	
303. Did you provide financial support for your spouse while she went to/is at MWH for the current child?	1. Yes<br>2. No	

304. Did you avail food when your spouse and relatives are at MWHs for the current child?	1.Yes<br>2.No	
305. Did you look after the home and or children while your spouse is at MWHs for the current child?	1.Yes<br>2.No	**If no pass to question 307**
306. If yes to question 305, what kind of care did you give?	1.preparing of their foods<br>2.cleaning of their cloths and their body<br>3.other (specify)________________	
307. If no to question 305, who took care to them?	1.My parents/family<br>2.My adult girl<br>3.My neighbors<br>4.our community health development armies<br>5.other (specify)	
308. Did you arrange transport when your spouse went to MWHs for the current child?	1.Yes<br>2.No	

Section 4 spousal obstetric history

**Questions**	**Responses**	**Skip rule**

401. Did your spouse give birth before the current child?	1.yes<br>2.no	**If no pass to question 408**
402. Did your spouse deliver at health facility before the current child?	1.yes<br>2.no	
403. Did your spouse have previous history of obstetric complication before the current child?	1.yes<br>2.no<br>3. I don't remember	**If no/didn't remember pass to question 405**
404. What kind of complication did occur?	1. Preterm labour<br>2. Premature rapture of membrane<br>3. Hemorrhage<br>4. Prolonged labor<br>5. Other (specify)	
405. Did your spouse face stillbirth before current birth?	1.yes<br>2.no	
406. How long did she stay at MWHs for the current child?	____________Days	
407. Did your spouse have ANC follow up for the current child?	1.yes<br>2.no	**If no pass to next section**

408. Did you accompany your spouse during current pregnancy for ANC?	1.yes<br>2.no	
409. Did you get counseling about MWH during visit?	1.yes<br>2.no	

Section 5. health facility related factors

Questions	Responses			Skip rule
501.Did the MWH had basic social service such as:<br>Sleeping space?<br>Beds?<br>Mattresses?<br>Extra spaces for accompanying families?<br>Bathroom?<br>Blankets?<br>Water?<br>Toilets?	Items	Yes	No	
	Sleeping space?			
	Beds?			
	Mattresses?			
	Extra spaces for accompanying families?			
	Bathroom?			
	Blankets?			
	Water?			
	Separate cooking area?			

Entertainment/recreational facilities (TV/Radio)?<br><br>Separate cooking area?	Entertainment/recreational facilities (TV/Radio)?			
	Toilets?			
502.Did the health center has ambulance for referral if complication occur	1.Yes<br><br>2.No			
503.Did the health care staff made close follow up(daily round ,take vital sign ,follow fetal condition by preparing fetal kick chart)	1.Yes<br><br>2.No			
504. How many hours take to reach MWH facility (on foot travel in minutes)?	-- -			

Section 6.Gender related factors affecting male partners' involvement in maternity waiting home utilization

**Questions**	**Responses**	**Skip rule**
601. What are gender related reasons that make male partners not to involve decision to rest in maternity waiting home? (more than one answer is possible)	1.Child-birth is a woman's affairs that does not require men participation<br><br>2. Childbirth is natural phenomenon that should not be given much attention by males.	

	3.Accompanying wife to maternity waiting home for delivery is women's responsibility<br><br>4. I don't know	
602. Do you have other wife other than the wife that living with you?	1.yes<br><br>2.no	
603.In your family who is the most decision maker in any cases that needs decision	1. Respondent<br>2. Wife<br>3. Both equally<br>4. I don't know	

Section 7. Knowledge and Attitude Questions about maternity waiting home

**Questions**	**Responses**	**Skip rule**
701. what kind of pregnant mother admitted at MWHs (more than one answer is possible)	1. Women from remote areas which is difficult for ambulance service or far from health facility<br>2. >=38 week pregnant mother i.e. 15 day left for delivery<br>3. previous pregnancy/delivery problems (previous preterm labor, previous still birth ,previous cervical tear)	

	4. health professional decisions that a mother is at risk<br>5. I don’t know<br>6. Other (specify)____________	
702. What is the importance of using maternity waiting home? (more than one answer is possible)	1.Access to skilled care<br>2.prevent delay in getting emergency care if needed<br>3.Immediate treatment to the mother and baby<br>4.I don’t know	
703. Do you think that the use of maternity waiting homes is linked to the use of maternal health services?	1.yes<br>2.no	If no pass to question 706
704. If yes for Q.no. 703 what maternal health service does the mother will get? (more than one answer is possible)	1.immunization<br>2.early initiation of exclusive breast feeding<br>3.nutritional counseling<br>4.family planning<br>5.other (specify)	

705. For whom do you think maternity waiting home is important?	1.Mother alone<br>2.Baby alone<br>3.Both to the mother and baby	
706. Using maternity waiting home can help for early detection of complications during labor and child birth.	1.Strongly agree<br>2.Agree<br>3.Neutral<br>4.Disagree<br>5.Strongly disagree	
707. The approach of health professionals towards service provision is not good.	1.Strongly agree<br>2.Agree<br>3.Neutral<br>4.Disagree<br>5.Strongly disagree	
708. Using maternity waiting home decrease neonatal mortality.	1.Strongly agree<br>2.Agree<br>3.Neutral<br>4.Disagree	

	5.Strongly disagree	
709. Male partners who accompany his wife to maternity waiting home are "feminist".	1.Strongly agree<br>2.Agree<br>3.Neutral<br>4.Disagree<br>5.Strongly disagree	
710. Using maternity waiting home can help for utilization of other maternal health services.	1.Strongly agree<br>2.Agree<br>3.Neutral<br>4.Disagree<br>5.Strongly disagree	
711. I discourage the use of maternity waiting home .because the cause of death of mother during labor and child birth is evil.	1.Strongly agree<br>2.Agree<br>3.Neutral<br>4.Disagree<br>5.Strongly disagree	

712. Most of the time those mothers who rest in maternity waiting home are lazy.	1.Strongly agree<br><br>2.Agree<br><br>3.Neutral<br><br>4.Disagree<br><br>5.Strongly disagree	
713. Maternity waiting home use can improve maternal survival	1.Strongly agree<br><br>2.Agree<br><br>3.Neutral<br><br>4.Disagree<br><br>5.Strongly disagree	

Thank you!!!
