# Supplementary material for: Factors affecting men’s involvement in maternity waiting home utilization in North Achefer district, Northwest Ethiopia: A cross-sectional study
Source: PLoS One. 2022 Feb 10;17(2):e0263809. doi: 10.1371/journal.pone.0263809 (PMC8830716; doi:10.1371/journal.pone.0263809)
Supplement: Attachment — Submitted filename: Manuscript_Clean_Copy.docx [file pone.0263809.s004.docx]

**Factors affecting men’s involvement in maternity waiting home care in North Achefer district, Northwest Ethiopia: a cross-sectional study**

**Men’s involvement in maternity waiting home care**

Getachew Asmare^1^, Dabere Nigatu^2*^, Yamrot Debela^3^

**Authors’ Affiliation:**

^1^Department of Reproductive Health and Nutrition, School of Public Health, College of Medicine and Health Sciences, WolaitaSodo University, WolaitaSodo, Ethiopia

^2^Department of Reproductive Health and Population Studies, School of Public Health, College of Medicine and Health Sciences, Bahir Dar University, Bahir Dar, Ethiopia.

^3^Department of Health Promotion&Behavioral Science, School of Public Health, College of Medicine and Health Sciences, Bahir Dar University, Bahir Dar, Ethiopia

*Corresponding author

E-mail: [daberen@yahoo.com](mailto:daberen@yahoo.com) (DN)

**Abstract**

**Background:** Maternity waiting home is a direct strategy to improve newborn and maternal survival. The utilization of maternity waiting home, however, remains very low in Ethiopia. The involvement of men in maternal health programs is a key strategy to increase the utilization of various maternal health services, including maternity waiting home. Thus, the current study aimed to identify factors affecting men’s involvement in maternity waiting home care.

**Methods:**A community-based cross-sectional study was conducted from November 1^st^ to December 30^th^, 2018. Four hundred three (403) men were involved in the study. Data were analyzed by the statistical package for social science (SPSS) version 23. Independent predictors were identified by a multivariable logistic regression model. Adjusted odds ratios (AORs) with 95% confidence intervals (CIs) were reported.

**Results:**Men involvement in maternity waiting home care was 55.6% (50.71, 60.45). Age (AOR=0.88,95% CI=0.82-0.94), knowledge (AOR=4.75,95% CI=2.65-8.49), decision-making power (AOR=4.00,95% CI=1.38-11.57), and receiving counseling about maternity waiting home during spousal antenatal care visits (AOR=9.15, 95% CI=3.34-25.03) had statistically significant associations with men’s involvement in maternity waiting home care.

**Conclusions:** Nearly, half of male partners were involved in maternity waiting home care. Men’s age, knowledge, decision-making power, and receiving counseling were factors affecting their involvement in maternity waiting home care. Targeted interventions on increasing men’s knowledge about maternity waiting home and changing patriarchal thinking in society can improve men’s involvement in maternity waiting home care.

**Keywords:** Men involvement; Male partners;Maternity waiting home;Ethiopia

# Introduction

Global experiences show that more than 80% of maternal deaths could have been prevented by appropriate and timely interventions performed by skilled professionals in a conducive environment[1, 2].In 2016, only 26% of women in Ethiopia gave birth at health facilities. This rate is among the lowest in the world. The reasons for non-use include notions that facility delivery is not necessary or customary, as well as physical distance to the facility and lack of transportation[3]. Many years since maternity waiting home (MWH) is articulated as a direct strategy to increase health facility delivery and improve maternal and newborn survival[4, 5].Maternity waiting homesare residential facilities located near a hospital or a health center that allow pregnant women to wait for the onset of labor. Once labor starts, women move to the health facility so that they can be assisted by a skilled birth attendant[6]. Pregnant women from remote areas, women with gestational age greater or equal to 38 weeks, women with previous pregnancy/delivery problems (previous preterm labor, previous still birth, previous cervical tear), or women with known risk declared by health professionals are eligible for accommodation at MWHs[7, 8].In 2016, a survey conducted in four regions of Ethiopia reported that 70% of health centers had maternity waiting homes[9] and Nationally, about half of the facilities had maternitywaiting homes[10]

Although MWH started in the late 1980s in Ethiopia[6], service uptake remains low because of sociodemographic, economic, cultural, gender- and facility-related constraints[7, 9]. Moreover, in most developing countries, including Ethiopia, most communities give a low position to women, which puts them either under collective decision making with their partner or completely dependson their partner decision on issuesthat affect their health[11].To overcome such problems, nearly two decades before, the concept of male involvement in maternal health has been promoted as an essential element of the World Health Organization initiative for making pregnancy and childbirth safer[12].The rationale for seeking to involve men includes a view of men as gatekeepers and decision-makers for prompt access to health services,as responsible partners of women,as an important member of the community,and men’s preference involve as fathers/partners[13].For example, 55% of women in Ethiopia to 95% of women in Kenya need their husband’s permission to use MWH[14, 15], while approximately one-third (33%) of mothers in Ethiopia experienced refusal of admission by husbands[9]. We found that studying men’s involvement in maternity waiting home use has paramount importance for policy-makers, programmers and health care planners to design evidence-based interventions. Therefore, this study aimed to determine the extent of male involvement in maternity waiting home care and identify the factors that affect their involvement in Northwest Ethiopia.

**Methods**

**Study design and settings**

A community-based cross-sectional study was conducted in the North Achefer district from October 1^st^, 2018 to December 30^th^, 2018. The district is located in the West Gojjam Zone, Amhara regional state, Ethiopia. It has a total of 27 kebeles (“kebele” is the lowest administrative unit in Ethiopia). Regarding health infrastructure, it has one primary hospital, seven health centers, five private clinics and twenty-seven health posts. During the time of data collection, there were seven maternity waiting homes at each health center in the district, but only five of the health centers had functional maternity waiting homes [16].

This research paper is prepared following the “Strengthening the Reporting of Observational Studies in Epidemiology (STROBE)” checklist for cross-sectional study reporting guidelines[17](S1 Table).

**Sample size and sampling procedure**

Primarily, we proposed including 442 male partners in the study, while 403 male partners were involved at the end. A single population proportion formulawas used to determine the sample size with the assumption of a 95% confidencelevel, 50% expected proportion of men involved, and 5% margin of error. The formula:

$$\mathbf{n=}\frac{\left( \mathbf{Z}_{\frac{\boldsymbol{\alpha}}{\mathbf{2}}} \right)^{\mathbf{2}}\mathbf{p(1-p)}}{\mathbf{d}^{\mathbf{2}}}\mathbf{=}\frac{\left( \mathbf{1.96} \right)^{\mathbf{2}}\boldsymbol{\times0.5}\left( \left. \mathbf{0.5} \right) \right.}{\left( \mathbf{0.05} \right)^{\mathbf{2}}}\mathbf{=384}$$

Where; n is the sample size, Z_α/2_ is critical value for normal distribution at 95% confidence level,p is the expected proportion, and d is the margin of error. We targeted to involve 442 men with consideration of 15% for non-responses. In the North Achefer district, only five health centers had functional maternity waiting homes. First, the principal investigator identified 662 mothers who hadused MWH in the last year from the maternity-waiting-home-users registration book. Then, residential profiles(kebeles and gotts) of mothers were identified from maternity waiting home registration books. Finally, mothers were selected by table of random numbers. Maternity-home-user mothers from nearby districts and whom husbands diseased or divorced were excluded. Males who were currently living with their spouse were considered in the study.

**Study variables and measurements**

Male partners were interviewed using a structured Amharic version questionnaire. We reviewed various literature to develop the questionnaire. The questionnaire was pretested on 5% of the target sample size. The pretest was done in the nearby district, designated as South Achefer district. Then, the questionnaire was amended for wordings, sequencing and contents as the pretest output suggested. We have attached the questionnaire both the Amharic and the English versions as supporting information (S1 file and S2 file). Trained data collectors and supervisors were involved in the data collection process. The interviewswereconductedin the respondents’ residential houses. If the selected respondent was not available at the time of the first home visit, two revisits were made.

The questionnaire comprised sociodemographic variables (age, educational status, wealth index, occupation and number of children); participant’s spousal obstetric history (previous stillbirth, previous health facility delivery, length of stay at MWH, ANC follow-up, history of spousal obstetric complication); health facility-related variables (basic socialservices, presence of ambulance, and daily follow-up at MWHs); male partner’s gender thinking (number of wives, decision-making power); and male partner’s knowledge and attitude towards MWH. Five knowledge items were used to assess men’s knowledge about maternity waiting home. All correct responses on five items were added to produce a composite index. We used eight items with a five pointLikert scale to assess men’s attitudes towards maternity waiting home. The sum score was generated by adding individual scoreson each item. Those men who scoredabove the median were considered to have a positive attitude towards maternity waiting home utilization otherwise taken as having a negative attitude.

The outcome variable for this study was men’s involvement inmaternity waiting home care. Six items were used to measure men’s involvement in maternity waiting home care. The items used include male partner participation in the decision to rest their spouse to MWH, accompanying spouse while she went to MWH, providingfinancial support while spouses went to/at maternity waiting home, availing food while spouses were at MWH and looked after the home and/or the remaining children while the spouses were at MWHs. Each item had yes or no response options and coded 1 yes or 0 no. We added the score on each item to generate a composite index for male involvement in MWH utilization. Those men who scoredless than three were considered as poor male involvement while those who scoredgreater than or equal to three were considered good male involvement.

**Data analysis**

Data were checked, coded and entered into Epi-data version 3.1 and exported to SPSS version 23 for analysis. The reliability of items used to measure male partners’involvement in MWH utilization, male partners’knowledge and attitudestowards MWH were checked by Cronbach’s alpha value. The Cronbach’s alpha value of the six items used to assess male partners’ involvement in maternity waiting home utilization was 0.73, which is at acceptable range. Binary logistic regression analysis was used to determine the association between explanatory variables and male partners’ involvement in maternity waiting home utilization. Those candidate variables thatwere significant (p<0.25) in the bivariable analysis were entered into the multivariable logistic regression analysis. Finally, adjusted odds ratioswith 95% confidence intervals wereused to identify independent predictors of men’s involvement in maternity waiting home utilization.

**Ethical considerations**

Ethical clearance letterswereobtained from the Bahir Dar University College of Medicine and Health Sciences Institutional Review Board. The permission letter was obtained from the North Achefer district administrative. Moreover, all the study participants were informed about the purpose and benefit of the study along with their right to refuse. The data collectors read the information sheet and consent form to each study participantuntil they comprehend the contents. Then, the participants were supposed to show their agreement or disagreement verbally instead of hand signed consent approval. Finally, the data collectors are supposed to circle on the appropriate response of the participant to proceed to the next step. The study participants were reassured to attain confidentiality. We maintained anonymity and confidentiality of information throughout the study process.

# Results

**Sociodemographic and economic characteristics of the study participants**

Four hundred three male partners were involved in the study,resulting ina response rate of 91.2%. Thirty-seven percent of males were between the age groups of 40-49. The majority, 96.8%, of males were orthodox Christian followers. Ninety-two percent were Amhara by ethnicity. Eighty-one percent of males were farmers. Approximately49% of males were unable to read and write. Nearly twenty-two males were rich and 20.8% were poor (Table 1).

**Table 1. Sociodemographic characteristics of study participants in North Achefer district, Northwest Ethiopia, 2018**

| **Variables** | **Frequency** | **Percentage** |
| --- | --- | --- |
| **Age category** | | |
| 18-29 | 77 | 19.1 |
| 30-39 | 91 | 22.6 |
| 40-49 | 149 | 37.0 |
| >=50 | 86 | 21.3 |
| **Educational status** | | |
| Unable to read and write | 196 | 48.6 |
| Read and write only | 82 | 20.3 |
| Primary education | 65 | 16.1 |
| Secondary education | 12 | 3.1 |
| Higher education | 48 | 11.9 |
| **Occupation** | | |
| Farmer | 326 | 80.9 |
| Merchant | 27 | 6.7 |
| Government employee | 50 | 12.4 |
| **Number of living children** | | |
| 1 | 94 | 23.3 |
| 2-4 | 138 | 34.3 |
| >=5 | 171 | 42.4 |
| **Wealth index quintile** | | |
| Very rich | 80 | 19.9 |
| Rich | 88 | 21.8 |
| Middle | 74 | 18.4 |
| Poor | 84 | 20.8 |
| Very poor | 77 | 19.1 |

**Obstetric histories of spouses of male partners**

Approximately seventy-seven per cent of their spouses gave birth before the current child. About 66.8% of their spouses had a previous history of health facility delivery, and 12.8% had a previous history of obstetric complications. The most common obstetric complications that occurred were hemorrhage (37.5%) and prolonged labor (37.5%). Eighty-six percent of respondents’ spouses stayed less than fifteen days at maternity waiting home for the current child. Eighty-two percent of respondents’ spouses had ANC follow-up for the current child, of whom 73% of men’s accompanied their spouse during ANC follow-up and 65.9% of male partners had received counseling during ANC about maternity waiting home (Table 2).

**Table 2. Study participant’s spousal obstetric history in North Achefer district, 2018**

| **Variables** | **Frequency** | **Percentage** |
| --- | --- | --- |
| **Previous delivery history** |  |  |
| Yes | 312 | 77.4 |
| No | 91 | 22.6 |
| **Previous health facility delivery** |  |  |
| Yes | 209 | 66.8 |
| No | 104 | 33.2 |
| **Previous history of obstetric complication** |  |  |
| Yes | 40 | 12.8 |
| No | 273 | 87.2 |
| **Types of obstetric complications** |  |  |
| Preterm labour | 2 | 5.0 |
| Premature rapture of membrane | 8 | 20.0 |
| Hemorrhage | 15 | 37.5 |
| Prolonged labor | 15 | 37.5 |
| **Previous still-birth history** |  |  |
| Yes | 11 | 3.5 |
| No | 302 | 96.5 |
| **Duration of stay at MWHs** |  |  |
| < 15 days | 347 | 86.1 |
| 15-30 days | 56 | 13.9 |
| **Spousal ANC visit** |  |  |
| Yes | 331 | 82.1 |
| No | 72 | 17.9 |
| **Male partner accompaniment during ANC visit** |  |  |
| Yes | 255 | 73.0 |
| No | 76 | 23.0 |
| **Male partners got counseling about MWH during 4^th^ visit** |  |  |
| Yes | 218 | 65.9 |
| No | 113 | 34.1 |

**Male partners’ gender thinking, knowledge and attitude towards MWH**

Male partners were asked about gender thinking that likely influencestheir involvement in resting pregnant women in maternity waiting home: 17.1% think that childbirth is women affairs that do not need the participation of males, 16.1% think that childbirth is a natural phenomenon that should not require males’ much attention, and 28.8% think that accompanying wife-to-maternity waiting home for delivery is women’s responsibility. Forty-four percent of men make decisionsalone in any case that needs decisionsin the family. Almost all, 99% of men involved in the study had only one wife (Table 3).

The Cronbach’s alpha value of the knowledge questions was 0.755. The Cronbach’s alpha value of the attitude questions was0.127.As indicated in Table 3,36.7%of respondents had a positive attitude.

**Table 3. Male partners’ gender thinking and attitude towards maternity waiting home in North Achefer district, 2018**

| **Variables** | **Frequency** | **Percent** |
| --- | --- | --- |
| **Child-birth is a woman’s affairs that does not require men participation** |  |  |
| Yes | 69 | 17.1 |
| No | 334 | 82.9 |
| **Child-birth is natural phenomenon that should not be given much attention by males** |  |  |
| Yes | 65 | 16.1 |
| No | 338 | 83.9 |
| **Accompanying wife to maternity waiting home for delivery is women’s responsibility** |  |  |
| Yes | 116 | 28.8 |
| No | 287 | 71.2 |
| **Who is the most decision maker in your family in any case that needs decision?** |  |  |
| Male alone | 179 | 44.4 |
| Wife alone | 50 | 12.4 |
| Spouses jointly | 174 | 43.2 |
| **Male partners having more than one wife other than the current wife living together** |  |  |
| Yes | 4 | 1.0 |
| No | 399 | 99.0 |
| **Attitude** |  |  |
| Positive attitude | 148 | 36.7 |
| Negative attitude | 255 | 63.3 |

**Men’s involvement in maternity waiting home**

The six items used to assess male partners’ involvement in maternity waiting home utilization wereevaluated by Cronbach’s alpha value. The Cronbach’s alpha was 0.727. The mean and median of the items were 3.26 and 4 respectively.

Overall, 55.6% of male partners had good involvement in maternity waiting home utilization (Fig1). The findingsof specific men’s involvement indicators shows that 56.3% of partners were decided to rest their spouse to MWH, 54.1% accompaniedtheir spouse to MWH, 52.6% providedfinancial support while their spouses went to/at maternity waiting home, 62.5% availedfood while spouses were at MWH and 45.9% looked after the home and/or the remaining children while their spouses were at MWH (Table 4).

**Fig 1. Overallmen’s involvement in maternity waiting home care, North Achefer district, 2018**

**Table4. Distribution of male partners’ involvement in maternity waiting home utilization in North Achefer district, 2018**

| **Variables** | **Frequency** | **Percent** |
| --- | --- | --- |
| **Decided their spouse to rest in MWH for current Child** |  |  |
| Yes | 227 | 56.3 |
| No | 176 | 43.7 |
| **Accompany their spouses for resting in MWH for current child** |  |  |
| Yes | 218 | 54.1 |
| No | 185 | 45.9 |
| **Provided financial support for their spouse while they went to/were at MWH for the current child** |  |  |
| Yes | 212 | 52.6 |
| No | 191 | 47.4 |
| **Availed food when their spouse and relatives were at MWHs** |  |  |
| Yes | 252 | 62.5 |
| No | 151 | 37.5 |
| **Looked after the home and or children while their spouses were at MWHs for the current child** |  |  |
| Yes | 185 | 45.9 |
| No | 218 | 54.1 |
| **Arranged transport when their spouse went to MWHs** |  |  |
| Yes | 218 | 54.1 |
| No | 185 | 45.9 |

**Factors influencing men’sinvolvement in maternity waiting home utilization**

To identify predictor variables of male partners’ involvement, we fitted a multivariable logistic regression model. In the bivariable logistic regression analysis, variables with p-valuesless than 0.25 were taken as candidate variables for the multivariable logistic regression model. Thus, age, occupation, educational status, number of live children, wealth index, distance from maternity waiting home, duration of stay at maternity waiting home, knowledge about maternity waiting home, attitude towards maternity waiting home, decision making power of respondents, spousal ANC follow-up, previous spousal health facility delivery, previous spousal obstetric complication, and receiving counseling about maternity waiting home during spousal ANC follow-up were entered into the multivariable model.

Based on findings from multivariable logistic regression analysis,a one-year increase in men’s age was associated with a 12% decrease in the likelihood of men involvement in maternity waiting home utilization (AOR= 0.88,95% CI= 0.82-0.94). Knowledge of male partners related to maternity waiting home was positively associated with involvement in maternity waiting home utilization.A unit increase in the total knowledge score was associated with a 4.75 timesincrease in the likelihood of involvement in maternity waiting home utilization (AOR=4.75, 95% CI=2.65-8.49). Husbands who mostly made decisions in casesthat needed decisionswere 4 times more likely to be involved in MWHs compared to those who decided equally after discussion (AOR=4.00,95% CI=1.38-11.57). Those male partnerswho received counseling about maternity waiting home during spousal ANC follow-up were 9times more likely involve in maternity waiting home utilization compared to those who had notreceived counseling(AOR=9.15, 95% CI=3.34-25.03) (Table5).

**Table5. Factors affecting male partners’ involvement in maternity waiting home utilization in North Achefer district, 2018**

| Variables | Male involvement | | COR(95% CI) | AOR(95% CI) |
| --- | --- | --- | --- | --- |
|  | Good | Poor |  |  |
| Age in year |  |  | 0.86(0.84, 0.89) | 0.86(0.82,0.94)** |
| Knowledge score |  |  | 8.57(5.29,13.87) | 4.74(2.65,8.49)** |
| Decision maker in family affair |  |  |  |  |
| Male alone | 31.8% | 12.7% | 2.18(1.41,3.40) | 4.00(1.38,11.57)* |
| Wife alone | 0.7% | 11.7% | 0.06(0.02,0.19) | 0.29(0.05,1.75) |
| Partners jointly | 23.1% | 20% | 1 | 1 |
| Received counseling about MWH |  |  |  |  |
| Yes | 55% | 10.9% | 19.78(11.07,35.36) | 9.15(3.34,25.03)** |
| No | 6.9% | 27.2% | 1 | 1 |

*COR crude odds ratio, AOR adjusted odds ratio, * indicatesvariables thatare significant at p<0.05, ** indicatesvariables thatare significant at p<0.001*

# Discussion

The study revealed that men involvement in maternity waiting home care was 55.6% with 95% CI (50.71-60.45).Our study also identified that age, knowledge towards MWH, decision making autonomy and receiving counseling were factors significantly influencing men’sinvolvement in maternity waiting home utilization.

This study revealed a low proportion of men are involved in maternity waiting home.It is assumed low because women’s are stayingin MWH until labour starts, which may take a long time (a few days to many weeks).A study performed in Ethiopia reported that on average, they stayed 14.8 days at the MWHs, and approximately40% of pregnant women stayed for two or more weeks[9].If a higher level of men involvement is not achieved than the level we obtained now, it likely compromises future use. The lack of men’s support in their longer stay at MWH would become bad experience for women to have an intention to use or to recommend for others.

This study identified that being younger is associated with increasedinvolvement in maternity waiting home utilization. This might be due to the changing caring attitude of younger men for their wife and would be born child and the reduced influence of patriarchal thinking among this age group compared to older men. This finding is consistent with a study performed in Lemoworeda of Ethiopia[18].

The current study found that men’s knowledge was associated with increased involvement in maternity waiting home utilization. Similarly, our study noted apositive association between receiving counseling about maternity waiting home during spousal ANC follow-up and male involvement in maternity waiting home utilization. This might be because having knowledge is a prerequisite to practice. If men have awareness ofthe benefit package of maternity waiting home through different outlets, including health worker counseling, they could be encouraged to be involvedin service uptake. This finding isconsistent with other studies doneto assessmen’sinvolvement in delivery servicesand in birth preparedness and complication readiness planin southern Ethiopia, Lemodistrictof Ethiopia, Ambo town of Ethiopia, Mekelletown of Ethiopia, Enderta district of Ethiopia, Kenya, India, Mali and Tanzania[14, 19-27].

Men alone decision making in a family decision was positively associated with male involvement in MWHuse. This finding implies the presence of male dominance in society. Thisfinding is supported by a qualitative study performedin Zambia that even though the decision-making process regarding utilization of MWHs mainly involved the husband and wife sitting down to discuss, the final decision was made by the husband after taking into consideration the potential risks of labour complications[19]. This might be assumed that whenever malesare the most decision makers in a family, they will have the power to allow/refuse their spouses to utilize maternal health services.

The study has limitation. The lack of standardized indicators/tools to measuremen’s involvement in maternity waiting home utilization may be a limitation of the study.

# **Conclusions**

Nearly half of male partners showed poor involvement in maternity waiting home utilization. Men’s knowledge towards MWH, receiving counseling during spousal ANC visits, men alone decision making in family affairs, and being younger age were factors positively influencingmen’s involvement in maternity waiting home utilization. Interventions targeting improving male involvement in maternity waiting home utilization shouldfocus on building men’s knowledge about MWH and changing patriarchal thinking in society.

# **List of abbreviations**

ANC: Antenatal Care; AOR: adjusted odds ratio; CI: confidence intervals; COR: crude odds ratio,MWH: Maternity Waiting Home; SPSS: Statistical Package for Social Science.

# Acknowledgment

We are very grateful to Bahir Dar University for giving us ethical clearance. We are also indebted to thank the North Achefer district health office, kebeleadministrations and study participants for their cooperation during data collection.

# References

1.	World Health Organization(WHO). Making pregnancy safer : the critical role of the skilled attendant : a joint statement by WHO, ICM and FIGO. Making pregnancy safer; Geneva: World Health Organization, Department of Reproductive Health and Research; 2004.

2.	Renfrew MJ, Homer C, Downe S, Mcfadden A, Muir NK, Prentice T, et al. Midwifery: an executive summary for the Lancet’s series. The Lancet. 2014;384:1–8.

3.	CentralStatisticalAgency (CSA)[Ethiopia] and ICF-International. Ethiopia Demographic and Health Survey 2016. Addis Ababa, Ethiopia and Rockville, Maryland, USA: 2016.

4.	Figa'-Talamanca I. Maternal mortality and the problem of accessibility to obstetric care; the strategy of maternity waiting homes. Social Science & Medicine. 1996;42(10):1381-90.

5.	Poovan P, Kifle F, Kwast BE. A maternity waiting home reduces obstetric catastrophes. World Health Forum. 1990;11:440-5.

6.	WHO. Maternity Waiting Homes: A review of experiences. Geneva: WHO, 1996.

7.	A G, L P, KW S. Maternity waiting homes in Ethiopia –three decades experience. Ethiop Med J. 2012;50(3):209–19.

8.	Federal Ministry Health of Ethiopia. Guideline for maternity waiting home utilization. 2017.

9.	Tadele G, Wasie B. Maternity waiting homes in Rural Health Centers of Ethiop: The situation, women’s experiences and challenges. Ethiopian Journal of Health Development. 2016;30(1):20-6.

10.	Tiruneh GT, Getu YN, Abdukie MA, Eba GG, Keyes E, Bailey PE. Distribution of maternity waiting homes and their correlation with perinatal mortality and direct obstetric complication rates in Ethiopia. BMC Pregnancy and Childbirth. 2019;19(1):214. doi: 10.1186/s12884-019-2356-x.

11.	Haile A, Enqueselassie F. Influence of Women's autonomy on couple's contraception use in Jimma town, Ethiopa2009.

12.	WHO. Programming for male involvement in reproductive health: Report of the meeting of WHO regional advisers in reproductive health WHO/PAHO, Washington, DC, USA, 5-7 September 2001. Geneva, Switzerland: WHO, 2002.

13.	WHO. WHO recommendations on health promotion interventions for maternal and newborn health 2015. Geneva, Switzerland: 2015.

14.	Mramba L, Nassir FA, Ondieki C, Kimanga D. Reasons for low utilization of a maternity waiting home in rural Kenya. International Journal of Gynecology & Obstetrics. 2010;108(2):152-3.

15.	Endalew GB, Gebretsadik LA, Gizaw AT. Intention to use Maternity Waiting Home among Pregnant Women in Jimma District, Southwest Ethiopia. Global Journal of Medical Research. 2016;16(6).

16.	Noth Achefer Woreda Health Office. North Achefer woredas health office biannual report. In: health mac, editor. 2018.

17.	von Elm E, Altman DG, Egger M, Pocock SJ, Gøtzsche PC, Vandenbroucke JP, et al. The Strengthening the Reporting of Observational Studies in Epidemiology (STROBE) Statement: Guidelines for Reporting Observational Studies. PLOS Medicine. 2007;4(10):e296. doi: 10.1371/journal.pmed.0040296.

18.	Nuradin A. assessment of male partners’ involvement in promoting skilled delivery attendance of spouses and associated factors in Lemo woreda, Hadiya zone, southern nation nationality people region Ethiopia. 2014

19.	Sialubanje C, Massar K, Kirch EM, Pijl MSGvd, Hamer DH, Ruiter RAC. Husbands‟ experiences and perceptions regarding the use of maternity waiting homes in rural Zambia. International Journal of Gynecology and Obstetrics. 2015;133(1):108-11.

20.	Demissie DB, Bulto GA, Terfassa TG. Involvement of Male in Antenatal Care, Birth Preparedness and Complication Readinessand Associated Factors in Ambo Town, Ethiopia. Health, Medicine and Nursing. 2016;27.

21.	Weldearegay HG. Determinant Factors of Male Involvement in Birth Preparedness and Complication Readiness at Mekelle Town; a community Based Study. Science Journal of Public Health. 2015;3(2):175-80. doi: doi: 10.11648/j.sjph.20150302.14.

22.	Nanjala M, Wamalwa D. Determinants of Male Partner Involvement in Promoting Deliveries by Skilled Attendants in Busia, Kenya. Global Journal of Health Science. 2012;4(2):60-7. doi: doi: 10.5539/gjhs.v4n2p60.

23.	Smith K, Dmytraczenko T, Mensah B, Sidibe O. Knowledge attitudes and practices related to maternal health in Bla Mali: results of a baseline survey. Bethesda, MD: The Partners for Health Reformplus Project, Abt Associates Inc., 2004.

24.	Chattopadhyay A. Men in maternal care: evidence from India. Journal of biosocial science. 2012;44(2):129-53.

25.	Vermeiden T, Braat F, Medhin G, Gaym A, Akker Tvd, Stekelenburg J. Factors associated with intended use of maternity waiting home in Southern Ethiopia. BMC Pregnancy and Childbirth. 2018;18(38). doi: DOI 10.1186/s12884-018-1670-z.

26.	Gebrehiwot H, Gebregziabher W, Gidey G. Assessment of Husbands’ Participation on Birth Preparedness and Complication Readiness in Enderta Woreda, Tigray Region, Ethiopia, 2012. Journal of Women’s Health Care. 2013;3(140). doi: doi:10.4172/2167-0420.1000140.

27.	Gibore NS, Ezekiel MJ, Meremo A, Munyogwa MJ, Kibusi SM. Determinants of Men's Involvement in Maternity Care in Dodoma Region, Central Tanzania. Journal of pregnancy. 2019;2019:7637124. Epub 2019/07/06. doi: 10.1155/2019/7637124. PubMed PMID: 31275654; PubMed Central PMCID: PMCPMC6582798.

**Supporting information**

**S1 Table: STROBE 2007 (v4) Statement—Checklist of items that should be included in reports of cross-sectional studies**

**S1 File: Amharic language version questionnaire**

**S2 File: English language version questionnaire**
