# Supplementary material for: Factors affecting men’s involvement in maternity waiting home utilization in North Achefer district, Northwest Ethiopia: A cross-sectional study
Source: PLoS One. 2022 Feb 10;17(2):e0263809. doi: 10.1371/journal.pone.0263809 (PMC8830716; doi:10.1371/journal.pone.0263809)
Supplement: Attachment — Submitted filename: Response to Reviewers.docx [file pone.0263809.s005.docx]

**Response to Academic Editor**

**Comment:** Dear authors on your scholarly work; you have brought an important study problem in the area of practice. However, the manuscript has multiple language usage flaws including punctuations, wordings, spelling and mainly grammar errors. These problems are found throughout the manuscript. Moreover, there are several methodological limitations as the reviewers raised. Therefore, please make repeated proofreading and thorough copyediting before resubmitting the manuscript. This would help increase the readability of the manuscript if published.

***Response:*** *We tried to edit the entire document of the manuscript. We edited the spelling, space between words, and the whole grammar as much as possible with online grammar and language checkers (Quill bot online checker and Scribendi costumer service). The methodological concerns raised by the reviewers were adequately explained in the point-by-point response to reviewers.*

**Response to reviewers**

**Response to reviewer #2**

**Comment #1**: There are still a few grammatical errors. Some suggested changes are highlighted here:

a. Introduction, page 4, lines 76-77: “Thus, the current study applied an inclusive definition for male involvement in MVH, which includes male participation in deciding to rest their spouse to MWH…”

b. Methods, page 6, lines 122-123: “Then, the questionnaire was amended for wording, sequencing, and content…”

c. Methods, page 7, line 149: “The items used include male partner participation in deciding to rest their spouse to MWH…”

d. Results, page 12, line 208: “…should not require much attention from men…”

e. Discussion, page 18, line 285 and conclusion, page 18, line 301: “Men’s sole decision-making in family affairs…”

f. Discussion, page 18, line 289: “Men being primary drivers…”

g. Discussion, page 18, line 293: “The study has limitations.”

***Response #1:*** *Thank you for suggesting editorial changes. We have revised the manuscript as per the comments.*

**Comment #2**: Results: a. Typically, the sample size (n=403) is placed inside the table, for example under “frequency”, versus in the table title.

***Response #2****: We have revised each table so as to include the sample size for each variable.*

**Comment #3**: Discussion: a. Page 17, line 270-273: When you say younger men/this age group, which age group are you referring to? In the results it says that a year increase in age was associated with a 14% decrease in the likelihood of men’s involvement – did you also do the multivariable logistic regression model by the age categories in Table 1?

***Response #3a****: Thank you for your deep insight about the variable age. Actually, we considered age as a continuous variable in the logistic regression model. Hence, the reviewer concern is very practical that we have revised the discussion in way it suits the result.*

b. Page 18, study limitations: Thank you for addressing my suggestion to discuss additional limitations of the study, specifically the generalizability of the study. However, when I say generalizability, I meant the degree to which your study findings are generalizable to other contexts outside North Achefer District or outside Ethiopia. So, when you say “this study is generalizable to male partners whose spouse have used MWH…” is it only generalizable to North Achefer District, throughout Ethiopia, or can it be generalized to other similar contexts?

***Response #3b****: Thank you for the comment. In fact, since the study follows scientifically sound methods, the findings are generatable to other settings in Ethiopia and outside Ethiopia with similar contexts. We have incorporated your concern in the updated version of the manuscript.*

**Response to reviewer#3**

**Comment #1**: The manuscript titled, “Factors affecting men’s involvement in maternity waiting home care in North Achefer district, Northwest Ethiopia: a cross-sectional study” still requires additional copy editing for English grammar and language.

***Response #1****: We have revised the whole manuscript for grammar and language.*

**Comment #2**: Update the abstract to include the definition of “male involvement” in the current study.

Also elaborate in the last sentence of the abstract to propose how to change “patriarchal thinking in society” to improve men’s involvement.

Authors state that the current study aimed to identify factors affecting men’s involvement in maternity waiting home care. Please define MWH care? Do the authors mean MWH use?

***Response #2****: Thank you for the comment. The abstract is revised to include reviewer’s suggestions. Regarding MWH care Vs MWH use, the authors’ intention was to say MWH use. Thus, we have replaced the word “care” by “use” in the updated version of the manuscript. In the current revision, the tern “use” is replaced by “utilization” in the whole document for consistency and more reflectiveness.*

**Comment #3:** Study variables and measurements: More details need to be provided for how the survey tool was developed. There are several questions in the supplementary file that are not addressed in the presentation of results. Variables remain poorly defined. Were responses to questions in Sections 5,6,7 open ended or multiple choice? More details are needed about how Likert scales were determined and tested.

***Response #2****: Thank you for the comment. The survey tool was developed by reviewing different relevant literature. We have also done a pretest before the actual survey and we have amended the survey tool as per the feedback received during the pretest. In fact, there may be data assessed by the survey tool but not addressed in the results. However, for sure, we believe that we have addressed all the results that are relevant to the objective of the current study.*

*The responses to questions in section 5, 6 and 7 were multiple choice. In some cases, more than one answer is possible and questions with such response options were clearly indicated in parenthesis.*

*Upon literature review, there are studies employing different point Likert scales: two-point scale, three-point scale, four-point scale, five-point scale, seven-point scale and even more than seven-point Likert scale (*Chang, 1997*,* Vagias, 2006*,* Taherdoost, 2019*). The use of below five-point scale could be very conservative or provide restrictive options for raters to choose while the use of seven or more than seven-point scale make rating complex and get sophisticate raters and usually recommended for well-educated raters. Literature also suggests that five-point scale appears to be less confusing and to increase response rate. It is also quite simple for interviewer to read out the complete list of scale descriptors as compared to seven-point or more than seven point Likert scales (*Bouranta et al., 2009*,* Dawes, 2008*). Thus, we preferred to use five-point scale since it provides freedom for raters and because of simplicity for rating. Additionally, the current study is a community-based study. In community-based studies where you can find people with diverse level of literacy, the use of a 7-point scale or more than a 7-point scale is not recommended. At the end, the pretest has helped us to check and decide about the understandability and simplicity a five-point Likert scale for raters and interviewers.*

**Comment #4:** Results: Justify the use of so many variables in one multivariable model. Provide rationale for how attitudes were labeled positive or negative. Several results had very wide confidence intervals and potential reasons for the wide intervals need to be offered in the text not only as response to review comments.

*The number of variables used in multivariable model were 14. As inclusion criteria, we have used p-value less than 0.25 as cut value to select candidate variables for multivariable regression model. The sample size is also adequate to do so based on the assumption that 10-20 observations per variable considered in the multivariable regression. For example, if we calculate required minimum sample size by taking the upper bound of the assumption (i.e., 20 observations per variable), it will give us 280 (14*20), which is less than the actual sample size.*

*Regarding the rationale for labeling positive or negative attitude, as a matter of fact, we can find studies that label attitude as favorable or unfavorable and at the same time, we can find studies that label attitude as positive or negative by using attitude mean score or median score. In this study, eight items with a five-point Likert scale were used to assess men’s attitudes towards MWH. Hence, those men who scored above the median were considered to have positive attitude towards MWH use otherwise taken as having negative attitude (*Fang et al., 2021*,* Seid and Hussen, 2018*,* Dahake and Shinde, 2020*). The rational for labeling attitude positive or negative is just to show inclination towards certain behavior. In the case of this study, positive attitude towards MWH utilization means they have inclination to use MWH while negative attitude reflects the behavioral inclination not to use MWH.*

*Potential reasons for wide confidence interval may include sample size, multicollinearity, and presence of cells with observation very closer to zero. In this study, multicollinearity was checked by variance inflation factor and had no role for the wide confidence intervals observed in the study. Still, the wide confidence intervals might be associated with sample size adequacy and cell with small observation. For example, the variable decision-making in family affair had one cell with small observation. Having said these all, the authors believe that the confidence intervals recorded in this study are not worse to affect the conclusions.*

**Reference**

BOURANTA, N., CHITIRIS, L. & PARAVANTIS, J. 2009. The relationship between internal and external service quality. *International Journal of Contemporary Hospitality Management,* 21**,** 275-293.

CHANG, L. 1997. Dependability of anchoring labels of Likert-type scales. *Educational and Psychological Measurement,* 57**,** 800-807.

DAHAKE, S. & SHINDE, R. 2020. Exploring Husband's Attitude Towards Involvement in his Wife's Antenatal Care in Urban Slum Community of Mumbai. *Indian journal of community medicine : official publication of Indian Association of Preventive & Social Medicine,* 45**,** 320-322.

DAWES, J. 2008. Do data characteristics change according to the number of scale points used? An experiment using 5-point, 7-point and 10-point scales. *International journal of market research,* 50**,** 61-104.

FANG, Y., LIU, P. & GAO, Q. 2021. Assessment of Knowledge, Attitude, and Practice Toward COVID-19 in China: An Online Cross-Sectional Survey. *The American journal of tropical medicine and hygiene,* 104**,** 1461-1471.

SEID, M. A. & HUSSEN, M. S. 2018. Knowledge and attitude towards antimicrobial resistance among final year undergraduate paramedical students at University of Gondar, Ethiopia. *BMC Infectious Diseases,* 18**,** 312.

TAHERDOOST, H. 2019. What Is the Best Response Scale for Survey and Questionnaire Design; Review of Different Lengths of Rating Scale / Attitude Scale / Likert Scale. *International Journal of Academic Research in Management (IJARM),* 8.

VAGIAS, W. M. 2006. Likert-type scale response anchors. *Clemson International Institute for Tourism & Research Development, Department of Parks, Recreation and Tourism Management. Clemson University*.
